# The impact of Ki-67 index, squamous differentiation, and several clinicopathologic parameters on the recurrence of low and intermediate-risk endometrial cancer

**DOI:** 10.17305/bjbms.2020.5437

**Published:** 2021-10

**Authors:** Birol Ocak, Fatma Öz Atalay, Ahmet Bilgehan Sahin, Mine Ozsen, Bahar Dakiki, Seray Türe, Merve Mesohorli, Hikmet Utku Odman, Özgür Tanrıverdi, Gökhan Ocakoğlu, Mehmet Bayrak, Hakan Ozan, Candan Demiröz, Seda Sali, Sibel Oyucu Orhan, Adem Deligönül, Erdem Cubukçu, Türkkan Evrensel

**Affiliations:** 1 Department of Medical Oncology, Faculty of Medicine, Uludag University, Bursa, Turkey; 2 Department of Surgical Pathology, Faculty of Medicine, Uludag University, Bursa, Turkey; 3 Department of Internal Medicine, Faculty of Medicine, Uludag University, Bursa, Turkey; 4 Department of Medical Oncology, Faculty of Medicine, Sitki Kocman University, Mugla, Turkey; 5 Department of Biostatistics, Faculty of Medicine, Uludag University, Bursa, Turkey; 6 Department of Gynecologic Oncology, Faculty of Medicine, Uludag University, Bursa, Turkey; 7 Department of Radiation Oncology, Faculty of Medicine, Uludag University, Bursa, Turkey

**Keywords:** Low-intermediate risk, endometrioid endometrial carcinoma, recurrence, squamous differentiation, Ki-67

## Abstract

Endometrial endometrioid carcinoma (EEC) represents approximately 75-80% of endometrial carcinoma cases. Three hundred and thirty-six patients with EEC followed-up in the authors’ medical center between 2010 and 2018 were included in our study. Two hundred and seventy-two low and intermediate EEC patients were identified using the European Society for Medical Oncology criteria and confirmed by histopathological examination. Recurrence was reported in 17 of these patients. The study group consisted of patients with relapse. A control group of 51 patients was formed at a ratio of 3:1 according to age, stage, and grade, similar to that in the study group. Of the 17 patients with recurrent disease, 13 patients (76.5%) were Stage 1A, and 4 patients (23.5%) were Stage 1B. No significant difference was found in age, stage, and grade between the case and control groups (p > 0.05). Body mass index, parity, tumor size, lower uterine segment involvement, squamous differentiation (SqD), and Ki-67 index with p<0.25 in the univariate logistic regression analysis were included in the multivariate analysis. Ki-67 was statistically significant in multivariate analysis (p = 0.018); however, there was no statistical significance in SqD and other parameters. Our data suggest that the Ki-67 index rather than SqD needs to be assessed for recurrence in patients with low- and intermediate-risk EEC.

## INTRODUCTION

Endometrial cancer (EC) is the second most frequently encountered gynecological cancer worldwide, and endometrioid endometrial carcinoma (EEC) represents approximately 75-80% of patients with EC [1-3]. EC is categorized as low-, intermediate-, and high-risk according to the stage, tumor grade, and histological subtype [4]. According to the International Federation of Gynecology and Obstetrics (FIGO) staging for patients with EC, patients with Stage IA-Grade 1/2 EEC are classified as the low-risk group, and patients with Stage 1A-Grade 3 and Stage 1B-Grade 1/2 EEC are included in the intermediate-risk group [4].

Although many factors such as histological subtype, Grade 3 tumor, ≥50% myometrium invasion, lymphovascular invasion, lymph node metastasis, and tumor size >2 cm are considered risk factors of recurrence, low- and intermediate-risk ECs have an excellent prognosis, with recurrence rates of about 5-6% without adjuvant therapy [5,6].

The relevance of Ki-67 as a biomarker for EC remains uncertain in the literature [7]. Ki-67 expression was reported to be positively correlated with tumor grade in patients with EEC [8]. However, there is a lack of consensus regarding its prognostic value in EC [9]. In the study of Jiang et al. [10], an increased risk of recurrence was found in Stage 1-2 EC patients with a high Ki-67 index.

Squamous differentiation (SqD), which describes tumors from cell layers with intercellular bridges and distinct cell membranes with or without keratinization, has been reported in approximately 13-25% of patients with EC [11-13]. The prognostic value of SqD in patients with EC, first described by Ziano and Kurman in 1988 and used instead of definitions such as adenoacanthoma and adenosquamous carcinoma, is not yet clear in low- and intermediate-risk group EEC patients [11,14,15].

In this study, we aimed to investigate the prognostic effect of Ki-67 index and SqD on recurrence in low- and intermediate-risk EEC patients, as well as the status of other prognostic factors.

## MATERIALS AND METHODS

### Study population and data collection

For this retrospective study, the medical files of 336 patients with a histologically confirmed diagnosis of EEC between January 2010 and December 2018 in the Department of Medical Oncology of Bursa Uludag University Faculty of Medicine were reviewed. Among these patients, 272 patients with low-intermediate-risk EEC patients identified using the European Society for Medical Oncology (ESMO) criteria were included in the study [4].

**FIGURE 1:**
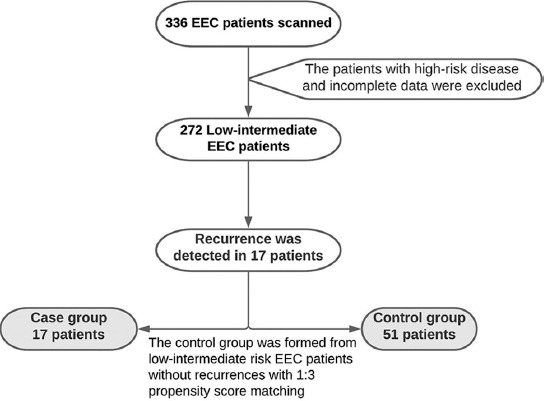
Diagram of the study design. EEC: Endometrioid endometrial carcinoma.

Only 17 of 272 patients with low and intermediate EEC included in the study had systemic or locoregional recurrence at any time during their follow-up, and these patients were named as the study group. Patients who did not develop any recurrence similar to the patients in the study group in terms of age, FIGO stage, and grade were classified as the control group by matching three to one ratio (Figure 1). Patients in both groups were compared in terms of demographic, clinical, and histopathological features, as well as SqD and Ki-67 levels.

Patients whose definitive surgical treatment was not performed in our institution, and those whose diagnosis of low and intermediate EEC could not be proven histopathologically, were excluded from the study. Surgical treatment of EC in our institution is a total hysterectomy and bilateral salpingo-oophorectomy. Intraoperative frozen section analysis was routinely performed in all cases. Pelvic and para-aortic lymphadenectomy is also performed for women whose frozen section analysis reveals a tumor type other than EEC, Grade 3 histology, cervical stromal invasion, myometrial invasion >50% depth, and tumor size >2 cm [16]. Brachytherapy was applied to the patients with Stage 1A/Grade 1-2 EEC, in the presence of high-risk factors (lymphovascular space invasion and age ≥60). Brachytherapy was applied to all patients to patients with Stage 1A/Grade 3 and Stage 1B. The treatment dose was given to the vaginal 1/3 apex area, 5 mm deep from the vaginal surface, with a high-dose rate brachytherapy device using the Ir-192 source. The doses applied to the vaginal mucosa, rectum, and bladder were calculated according to the International Commission on Radiation Units and Measurements. A total dose of 18-24 gray (Gy) was planned with a fraction dose of 6-7 Gy [16].

### Pathological assessment

Tumor size, histological type of tumor, grade, myometrial invasion, lymphovascular invasion, and lower uterine segment involvement were obtained from the patients’ pathology reports.

**FIGURE 2:**
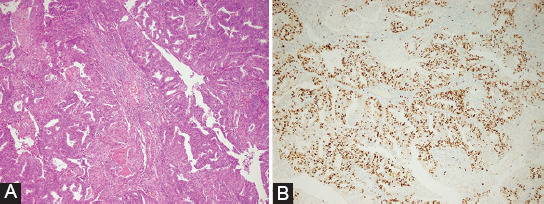
(A) Squamous differentiation, sheet-like growth without glands, sharp cellular margins, and glassy eosinophilic cytoplasm (H&E, ×10). (B) Ki-67 proliferation index is 60%.

SqD was proven by detecting at least three of the four criteria listed below, and SqD was expected to account for at least 10% of the tumor [12,17]. The criteria sought for SqD are listed as follows: Sheet-like growth without glands or palisading, sharp margins at a cell, thick and glassy eosinophilic cytoplasm, and nuclear/cytoplasmic ratio decreased more than foci elsewhere in the same tumor [12]. These criteria were used to detect SqD in reevaluations of each sample, and SqD was classified into two groups as either absent or present (Figure 2A).

Two independent pathologists reevaluated the Ki-67 proliferation index from immunohistochemically stained slides and SqD from hematoxylin-eosin stained slides. The slides of the cases were evaluated using a light microscope (model BX51TF, Olympus, Tokyo, Japan). The Ki-67 proliferation index was defined in the literature as the ratio of immunohistochemically stained nuclei to the total number of nuclei of tumor cells, independent of immunostaining intensity (weak, moderate, and strong) (possible range: 0-100%) [18]. At least 1000 cells were counted at ×400 from the hotspot areas of each sample in our study. Only nuclear staining was considered positive immunostaining, and the staining was scored according to the percentage of nuclear staining (Figure 2B). The Ki-67 assay clone used was 30-9.

### Ethical statement

Our study was conducted in accordance with the 1964 Helsinki Declaration. The clinical research ethics committee of the Bursa Uludag University Faculty of Medicine approved the study (Approval number: 2020-16/13).

### Statistical analysis

The Shapiro–Wilk test was used to assess whether the variables followed a normal distribution. Variables were reported as median (minimum–maximum) values. According to the normality test results, Mann–Whitney’s U-test was used to compare the groups. Categorical variables were compared using the Chi-square test and Fisher’s exact test, and 1:3 propensity score matching was performed to determine the control group (in terms of age, FIGO stage, and grade). Univariate logistic regression analysis was performed to determine independent risk factors. Any variable with p<0.25, according to the univariate logistic regression analysis, was accepted as a candidate for the multivariate model. The outcomes were compared using SPSS software (IBM Corp. Released 2017. IBM SPSS Statistics for Windows, Version 25.0, Armonk, NY: IBM Corp). A *p* < 0.05 was considered statistically significant.

## RESULTS

The comparison of the patients in the study group and the control group in terms of study variables is shown in Table 1. No significant difference was found in age, stage, and grade between both groups (*p* > 0.05). In addition, there was no statistically significant difference between the two groups in terms of body mass index (*p* = 0.079), the status of parity (*p* = 0.160), presence of myometrium invasion (*p* = 0.568), presence of lymphovascular space invasion (*p* = 0.355), median tumor diameter (*p* = 0.081), and lower uterine segment involvement (*p* = 0.095). There was also no statistically significant difference in lymph node dissection (*p* = 0.482) and adjuvant brachytherapy (*p* = 0.468).

**TABLE 1:**
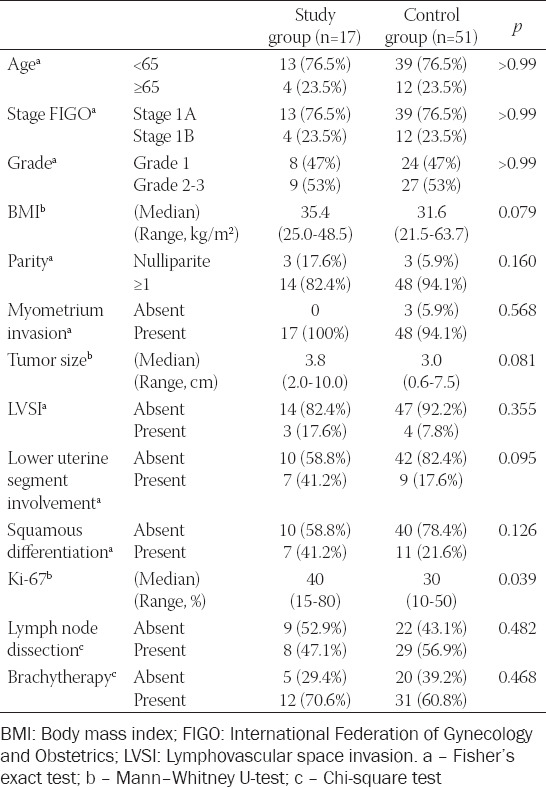
Univariate analysis of predictive recurrence for low- and intermediate-risk endometrioid endometrial cancer

SqD was observed in 7 patients (41.2%) in the study group and 11 (21.6%) patients in the control group. No significant difference was found in SqD between both groups (*p* = 0.126). The median ratio of Ki-67 was 40% (min–max range: 15-80) in the study group and 30% (min–max range: 10-50) in the control group, and it was statistically significant (*p* = 0.039).

Recurrences were reported 47.0% in vagina (8 patients), 23.5% in the lungs (4 patients), 5.9% in intra-abdominal lymph node (1 patient), 5.9% in peritoneum (1 patient), 5.9% in colon (1 patient), 5.9% in bone (1 patient), and 5.9% in bladder (1 patient).

After univariate logistic regression analysis, six parameters with p<0.25 were included in the multivariate logistic regression analysis: BMI, parity, tumor size, lower uterine segment involvement, SqD, and Ki-67 (Table 1). On multivariate analysis, only Ki-67 was significantly associated with recurrence (p = 0.018) (Table 2). The Ki-67 index had a 1.05-fold increased risk of recurrence in the low-intermediate-risk EEC study group than in the control group (Table 2).

**TABLE 2:**
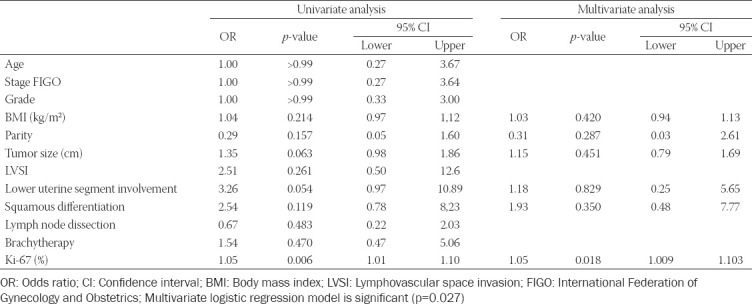
Univariate and multivariate logistic regression analysis of predictive recurrence for low- and intermediate-risk endometrioid endometrial cancer

## DISCUSSION

In our retrospective case–control study, we found that SqD did not significantly affect recurrence in our patients with low and intermediate EEC. In contrast, the Ki-67 proliferation index had a significant effect on recurrence. No statistical significance was found for other parameters such as body mass index, parity, tumor size, and lower uterine segment involvement.

The importance of SqD on recurrence in EC patients has been investigated by a limited number of studies in the current literature (Table 3) [13-15,19-22]. However, there have been different results regarding the effect of SqD on recurrence in both studies involving Stage 1 EC or Stage 1 EEC patients as well as patients with Stages 1-4 EC and/or Stages 1-4 EEC and other studies with low- and intermediate-risk EEC patients (Table 3) [13-15,19-22]. Although these studies contribute to the literature on the effect of SqD on recurrence, there is only one reported study to our knowledge, by De Andrade et al., investigating low-intermediate EEC patients [22].

**TABLE 3:**
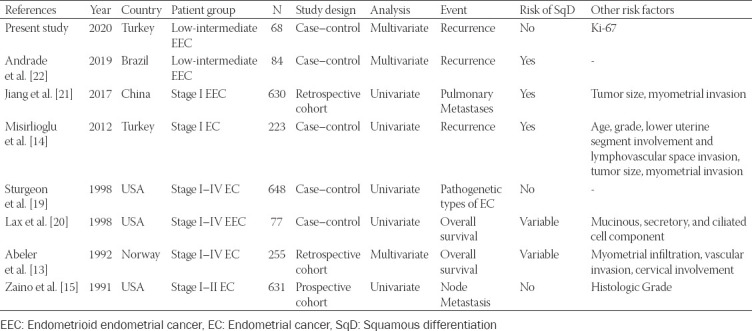
Summary of squamous differentiation endometrioid endometrial cancer studies to predict recurrence

In our study, we aimed to conduct a case–control study to investigate the effect of SqD on recurrence in low-intermediate-risk EEC patients. Unlike the study by De Andrade et al. [22], we formed the control group considering not only age and FIGO stage but also the grade. Additional risk factors such as myometrium invasion, lower uterine segment involvement, and Ki-67 index were included in the study.

The effect of SqD on recurrence in patients with EC remains controversial. In the study of Misirlioğlu et al. [14], statistical significance was found in univariate analysis in patients with EEC with SqD in the subgroup analysis of patients with EC. However, multivariate analysis was not performed in their study [14]. Although SqD was statistically significant in patients with pulmonary metastasis in the study by Jiang et al. [21] of patients with Stage 1 EEC, disease recurrence was not discussed in the study. By supporting our findings, the effect of SqD on recurrence in EC patients was not shown in the studies of Zaino et al. [15] and Sturgeon et al. [19].

Ki-67 protein is present during all active stages of the cell cycle (G1, S, G2, and M), excluding the resting cells (G0) [23]. It is used as a marker of cellular proliferation; its prognostic and predictive value was shown in several cancer types, including EC [24,25]. The effect of the Ki-67 index on recurrence, especially in low-intermediate EEC, has not been sufficiently investigated in the current studies. The Ki-67 index was not included in the study of Andrade et al. [22] investigating low-intermediate EEC patients. In the study of Jiang et al. [21] in patients with Stage 1 EEC, the effect of the Ki-67 index was not statistically significant on the development of pulmonary metastasis; however, the effect on disease recurrence was not reported. Yu et al. [26] examined patients with Stages 1-4 EC and found that Ki-67 was associated with stage, differentiation, depth of myometrium invasion, and lymph node status. In the study of Lax et al. [20] investigating Stages 1-4 EEC, the Ki-67 proliferation index was significantly higher in endometrioid carcinomas with a high-grade squamous component than the other tumors with various types of cellular differentiation and pure low-grade EEC. Therefore, the effect of SqD should be evaluated concurrently with Ki-67 in multivariate analysis. Some previous studies reported that a high Ki-67 index correlated with a high percentage of p53 stained immunohistochemically [27,28]. However, as we emphasized before, the results regarding the prognostic value of the Ki-67 index in low- and intermediate-risk endometrial carcinoma are not precise. Besides, there is literature information that p53 mutation is associated with cell differentiation in many cancers [29-31]. These studies revealed that cells without p53 mutation or carrying p53 activation tended to be well differentiated. Since our study is retrospective and the p53 was not evaluated in all patients, it may be considered to conduct new molecular-based studies to elucidate the relationship of SqD with the increased Ki-67 index according to the p53 mutation or activation.

## CONCLUSION

In the literature, studies investigating the effect of SqD on recurrence in low-intermediate-risk EEC patients are limited to the best of knowledge. In low-intermediate-risk EEC patients, SqD and Ki-67 index should be evaluated concurrently for recurrence. This study enabled us to compare the effect of SqD on recurrence with the literature. Determining factors affecting recurrence in low-intermediate-risk EEC patients may lead to changes in follow-up and treatment algorithms.
